# *In Vivo* Evaluation of Biocompatibility and Chondrogenic Potential of a Cell-Free Collagen-Based Scaffold

**DOI:** 10.3389/fphys.2017.00984

**Published:** 2017-11-29

**Authors:** Giovanna Calabrese, Rosario Gulino, Raffaella Giuffrida, Stefano Forte, Elisa Figallo, Claudia Fabbi, Lucia Salvatorelli, Lorenzo Memeo, Massimo Gulisano, Rosalba Parenti

**Affiliations:** ^1^Department of Biomedical and Biotechnological Sciences, University of Catania, Catania, Italy; ^2^Istituto Oncologico del Mediterraneo Ricerca, Catania, Italy; ^3^Fin-Ceramica Faenza, Faenza, Italy; ^4^Department of Medical and Surgical Sciences and Advanced Technologies, G.F. Ingrassia, “Policlinico Vittorio Emanuele”, Anatomic Pathology Section, University of Catania, Catania, Italy; ^5^Department of Experimental Oncology, Mediterranean Institute of Oncology, Viagrande, Italy

**Keywords:** tissue-engineering, scaffold, mesenchymal stem cells, chondrogenesis, cartilage regeneration

## Abstract

Injured articular cartilage has a limited innate regenerative capacity, due to the avascular nature and low cellularity of the tissue itself. Although several approaches have been proposed to repair the joint cartilage, none of them has proven to be effective. The absence of suitable therapeutic options has encouraged tissue-engineering approaches combining specific cell types and biomaterials. In the present work, we have evaluated the potential of a cell-free Collagen I-based scaffold to promote the augmentation of cartilage-like phenotype after subcutaneous implantation in the mouse. Forty female mice were grafted subcutaneously with scaffolds, while four additional mice without scaffold were used as negative controls. The effects of scaffold were evaluated at 1, 2, 4, 8, or 16 weeks after implantation. Immunohistochemical analysis shows the expression of typical cartilage markers, including type-II Collagen, Aggrecan, Matrilin-1 and Sox 9. These data are also confirmed by qRT-PCR that further show that both COL2A1 and COL1A1 increase over time, but the first one increases more rapidly, thus suggesting a typical cartilage-like address. Histological analysis shows the presence of some pericellular lacunae, after 8 and 16 weeks. Results suggest that this scaffold (i) is biocompatible *in vivo*, (ii) is able to recruit host cells (iii) induce chondrogenic differentiation of host cells. Such evidences suggest that this cell-free scaffold is promising and represents a potential approach for cartilage regeneration.

## Introduction

The regenerative capacity of articular cartilage after traumatic damage or disease is complicated by its avascular and relatively acellular nature (Lafont, 2010; Lam et al., 2015; Huang et al., 2016). Many therapeutic approaches have been developed for the treatment of cartilage damages, including osteo-chondral autografts and allografts, microfracture, autologous chondrocyte implantation and mesenchymal stem cell (MSC)-based therapies. However, these approaches have been often followed by significant donor site morbidity and by the formation of fibro-cartilaginous tissue that possesses biochemical and biomechanical feature definitely lower than that of natural hyaline cartilage (Roberts et al., 2003; Gobbi et al., 2005; Bae et al., 2006; Kreuz et al., 2006; Gomoll et al., 2010; Nukavarapu and Dorcemus, 2013).

Since many literature evidences reveals the lack of clinical solutions for osteo-chondral injury management, the field of cartilage repair is an area where innovative alternative therapy is needed (Phillips, 2005; Marcacci et al., 2013; Mithoefer, 2013; Lam et al., 2015). Based on these evidences, tissue engineering is the most promising alternative for repairing osteo-chondral damage. Novel tissue engineering strategies are focusing on the development of cartilaginous biomimetic materials able to repair cartilage lesions in association to cells and trophic factors (Chang et al., 2005; Moutos and Guilak, 2008; Liao et al., 2014; Calabrese et al., 2017a).

Wide varieties of synthetic or natural biomaterials have been employed so far to create scaffolds able to replicate the physicochemical properties of natural tissue (Stoppel et al., 2015). The scaffold offers a three-dimensional environment necessary to produce a functional cartilaginous matrix. The fundamental characteristic of the scaffold should be tolerability and biocompatibility, namely the capability of promoting adhesion, migration, growth, and differentiation of cells (Meyer et al., 2009).

Recently, our laboratory has evaluated, *in vitro*, the biocompatibility and the chondrogenic potential of a new 3D collagen I-based scaffold seeded with human adipose-derived MSCs (hADSCs) (Calabrese et al., 2017a). These cells have shown a good capacity of proliferation and multi-lineage differentiation, *in vitro*, confirming recent findings (Heydarkhan-Hagvall et al., 2008; Han et al., 2014; Calabrese et al., 2015; Vicari et al., 2016). The collagen scaffold has shown a high biocompatibility with hADSCs, as well as an intrinsic property of promoting hADSC chondrogenic differentiation, especially in presence of diffusible growth factors (Calabrese et al., 2017a). However, the intrinsic capacity of this material to integrate to the host, remain stable for long time without significant reactions of a immunocompetent animal, and promote cell recruiting and chondrogenic augmentation is still unknown and requires further studies. If confirmed, this putative capacity will allow the use of a cell-free biomimetic material without employing cell cultures or the use of exogenous growth factors.

The microstructural properties of this biomaterial, in term of density and elasticity, resemble those of cartilage and its composition allows coupling the high biocompatibility and biomimetic features to a simple and low-cost productive process (Deponti et al., 2014).

In the present work, we sought to characterize, *in vivo*, the biocompatibility and chondrogenic capacity of the same biomaterial after implantation in immunocompetent mice. The scaffold was subcutaneously implanted, cell-free, into the dorsum of mice and then analyzed at 1, 2, 4, 8, and 16 weeks after grafting, by *in vivo* imaging and histology to study the chondrogenic and angiogenic processes within the scaffold. Moreover, the host cells populating the biomaterial has been characterized by immunohistochemical analysis, to evaluate any inflammatory reaction and the expression of typical cartilage markers, including type-II Collagen, Aggrecan, Matrilin-1 and Sox 9.

## Materials and methods

### Scaffold features

3D collagen-based scaffolds used in this study have been produced by Fin-Ceramica Faenza SpA (Faenza, Italy). They have a cylindrical shape, with an 8 mm diameter and 5 mm height, consisting of equine type I Collagen gel (1 wt%) supplied in aqueous acetic buffer solution (pH = 3.5) (Opocrin SpA, Modena, Italy). The process of fabrication, as well as the chemical and physical characterization and tolerability have been described previously (Calabrese et al., 2017a). Briefly, collagen gel was softly dilute in sterilized water and precipitated in fibers by drop-wise addition of 0.1 M NaOH solution up to the isoelectric point (pH = 5.5). A crosslinking reaction was performed by 48 h-long immersion of the agglomerated fibers in NaHCO3/Na2CO3 (Sigma Aldrich and Merck Millipore) aqueous solution with a 1,4-butanediol diglycidyl ether (BDDGE) solution at 37°C to maintain scaffold structure. Then, agglomerated fibers were freeze-dried for 25 h under vacuum conditions (*P* = 0.29 mbar) to obtain a porous 3D structure. Finally, scaffolds were gamma-sterilized at 25 kGy. The microstructural and morphological characterization of scaffold was assessed by Scanning Electron Microscopy (SEM) by using a SEM-LEO 438 VP (Carl Zeiss AG, Oberkochen, Germany). The samples were sputter-coated with gold before analysis.

### Animals and experimental design

Female mice (*n* = 44) (BALB/cOlaHsd, 6 weeks aged, weight: 17–22 g; Harlan Laboratories) were used. Animal care and handling were performed according to EU Directive 2010/63/EU and the Italian law (D.Lgs. 26/2014). All experiments involving animals have been approved by the Italian Ministry of Health. Animals were housed in groups of four in independently ventilated cages (15 changes/hour of filtered air), with *ad libitum* access to water and food (Teklad rodent diet, Harlan Laboratories, San Pietro al Natisone, Italy), with standard conditions of temperature (22 ± 2°C) and relative humidity (50 ± 5%) and a light/dark cycle of 12/12 h.

Surgery was performed under aseptic conditions, maintaining mice under gas anesthesia (isoflurane). All efforts were made to minimize the number of animals used and their suffering. Pre- and post-grafting procedures were performed as explained previously (Calabrese et al., 2016b, 2017b). Briefly, surgical procedures were performed under aseptic conditions, with the animals under gas anesthesia (isoflurane). One collagen type-I scaffold/animal was implanted into a subcutaneous pocket in the dorsum of mouse. The transplanted mice were randomly divided in five groups: 1 week (*n* = 8), 2 week (*n* = 8), 4 week (*n* = 8), 8 week (*n* = 8), 16 week (*n* = 8), and finally sacrificed by intracardiac injection of Tanax (MSD Animal Health Srl, Segrate, Italy) under deep anesthesia (isoflurane). Four untreated animals were used as negative controls for *in vivo* imaging analysis. The scaffolds were explanted to perform *ex-vivo* analyses.

### Fluorescence molecular tomography (FMT) imaging *in vivo*

In order of assessing the occurrence of angiogenesis within the scaffold in a time-course manner, animals were evaluated by *in vivo* FMT (FMT 2500, Perkin Elmer, Monza, Italy). Specifically, all animals received an injection of 100 μl of AngioSense 680EX (Perkin Elmer, Monza, Italy) into the tail vein. This fluorescent probe specifically binds to endothelial cells. Twenty-four hours after the probe injection, FMT images were acquired. During the imaging, mice were maintained under isoflurane anesthesia. Acquisition and analysis of FMT images were assessed by using the TrueQuant software (Perkin Elmer, Monza, Italy). For quantification, the region of interest (ROI) was selected and the extent of angiogenesis was analyzed by measuring the amount of fluorescence probe (in pmol) into the ROI after choosing a concentration threshold. This threshold has been determined by keeping the volume of ROI constant (50 mm^3^). Animals were sacrificed by decapitation under deep anesthesia immediately after imaging.

### Histology

Immediately after animal sacrifice, scaffolds were collected and fixed for 2 h in 4% paraformaldehyde, dehydrated, embedded in paraffin and cut into 3 μm-thick sections. Sections were mounted on slides and treated for immunohistochemical analysis, as described previously (Calabrese et al., 2016a,b, 2017a,b). For immunofluorescence analysis the following rabbit polyclonal primary antibodies (LSBio, Seattle, WA, USA) were used: anti-Aggrecan (dilution: 1:150), anti-Matrilin-1 (1:200), anti-Sox9 (1:200), anti-type II Collagen (1:200). AlexaFluor anti-rabbit 568 was used as secondary antibody (dilution: 1:2000, Life Technologies) and nuclei were counterstained with DAPI (dilution: 1:10.000). Control of immunostaining specificity was assessed without the primary antibody. For immunohistochemistry analysis the mouse anti-CD15 (dilution: 1:100) monoclonal antibody (Abcam, Italy) was used. Washed sections were treated with biotinylated anti-mouse and anti-rabbit immunoglobulins and then incubated in streptavidin conjugated with peroxidase (Dako, Carpintera, Calif., USA). Staining was detected using diaminobenzidine (DAB). Sections were counterstained with hematoxylin, then dehydrated and mounted in permanent non-aqueous mounting medium.

Cell counts have been performed by using ImageJ software (NIH, USA). Data were analyzed as percentage of positive cells on the total number of DAPI stained cells. Three sections/animal have been used for counting.

For histological analysis alternate sections were also stained with Hematoxylin and Eosin (H&E), Alcian Blue (Panreac, Castellar del Valles, Barcelona, Spain) and Safranin O/Fast Green.

H&E staining was performed as previously reported (Calabrese et al., 2016a,b, 2017b). Alcian Blue staining was assessed according to the manufacturer protocol. The stained slides were examined by using a Leica DMI 4000B fluorescence microscope. A semi-quantitative evaluation of the formation of cartilage matrix has been performed by the measurement of optical density (OD) in gray scale images of the Alcian Blue stained specimens. Three images/animal have been randomly selected for OD quantification and measurements have been performed by using ImageJ.

For the staining a Safranin O solution was prepared according to manufacturer protocol. Sections were previously stained with Hematoxilin and Fast green, washed and incubated in 0.1% Safranin O solution for 5 min. Successively, slides were washed several times to get rid of the excess of the staining solution and, finally, mounted.

### Quantitative real time polymerase chain reaction (q-PCR)

Total RNA was isolated from scaffolds by using TRIzol reagent (Invitrogen Inc.). Reverse transcription of total RNA was carried out using High-Capacity cDNA Reverse Transcription Kit (Applied Biosystems). The transcriptional analysis of chondral genes, including COL1A1, COL2A1 e Aggrecan, was performed using SYBR Green method on a StepOne System Real Time PCR (Applied Biosystems). Primers for selected murine mRNA were designed using primer blast39 using exon-exon junctions on messengers as target region. The mRNA level of glyceraldehyde-3-phosphate dehydrogenase (GAPDH) and beta-tubulin (TuBB4a) were used as endogenous controls.

Primers sequences:

COL1A1_Forward: CCGGAAACAGACAAGCAACCCAAA;
COL1A1_Reverse: AAAGGAGCAGAAAGGGCAGCATTG;
COL2A1_Forward: TGGTCTTGGTGGAAACTTTGCTGC;
COL2A1_Reverse: AGGTTCACCAGGTTCACCAGGATT;
Aggrecan_Forward: TGTGGTGATGATCTGGCACGAGAA;
Aggrecan_Reverse: CGGCGGACAAATTAGATGCGGTT;
Sox9_Forward: AACAACCCGTCTACACACAGCTCA;
Sox9_Reverse: TGGGTAATGCGCTTGGATAGGTCA;
GAPDH_Forward: TGTGAACGGATTTGGCCGTA;
GAPDH_Reverse: ACTGTGCCGTTGAATTTGCC;
TuBB4a_Forward: GACGTGAGTACTGCTCCGC;
TuBB4a_Reverse: CTTGCAGGTGCACGATTTCC.

### Statistical analysis

Data were analyzed either as raw data or as mean ± SEM, as appropriate. Differences between groups were evaluated by using one-way ANOVA, followed by Tukey's HSD *post hoc* test. For all experiments, a *P* < 0.05 was considered to be significant. All analyses were assessed by means of Systat 11 (Systat Software, USA).

## Results

### Morphological characterization of scaffold

**Figure 1:**
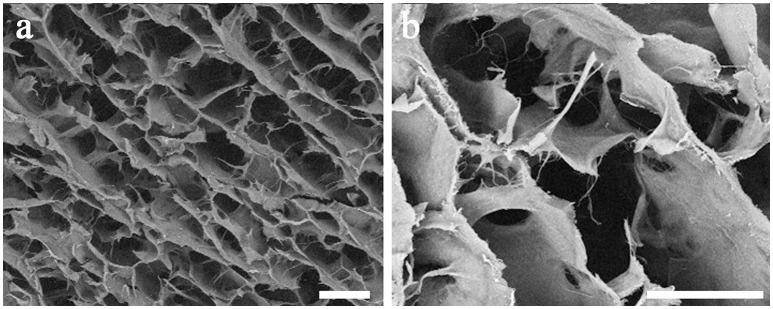
SEM images of the collagen-based scaffold. At higher magnification, interconnected collagen fibers are detectable within the scaffold. Magnification: 50x in **(a)**; 150x in **(b)**. Scale bars: 200 μm in **(a)**; 100 μm in **(b)**.

The morphological and microstructural features of scaffold were assessed by SEM analysis. Figure 1 reports SEM images of the scaffold at two different magnifications, 50x (Figure 1a) and 150x (Figure 1b). SEM images reveal an elevated porosity with three-dimensionally intersected pores without any precise alignment of the collagen fibers. This specific structure can be useful both for functions and for guiding cells during migration and proliferation.

### *In vivo* evaluation of new vascularization within the scaffold by FMT imaging

**Figure 2:**
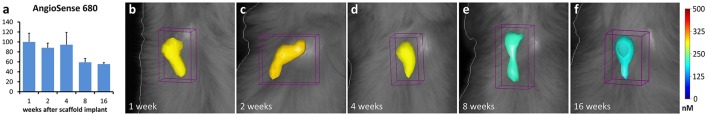
*In vivo* FMT quantification **(a)** and images **(b–f)** of angiogenesis within the implanted collagen-based scaffolds. The mean values calculated within groups were normalized to the mean value of 1-week group. The color scale indicates mean probe concentrations within the ROI.

The results of FMT analysis have demonstrated the presence of new vascularization within the implanted scaffolds, *in vivo*. Specifically, after intravenous injection of AngioSense 680, a strong fluorescent signal appeared in the anatomical area where the scaffold implant was placed. This signal was higher at the earlier time-point (1–4 weeks; Figures 2b–d), with a decreasing trend along the longer time-points (8–16 weeks; Figures 2e,f), although this decrease was not statistically significant (*P* > 0.05; Figure 2a).

### *Ex vivo* evaluation of cartilage developing within the scaffold

To assess the biocompatibility and capability of the new biomimetic 3D scaffold to promote augmentation of the cartilage-like tissue after implantation *in vivo*, we performed several histological analyses on the explanted scaffolds at 1, 2, 4, 8, and 16 weeks after implantation.

**Figure 3:**
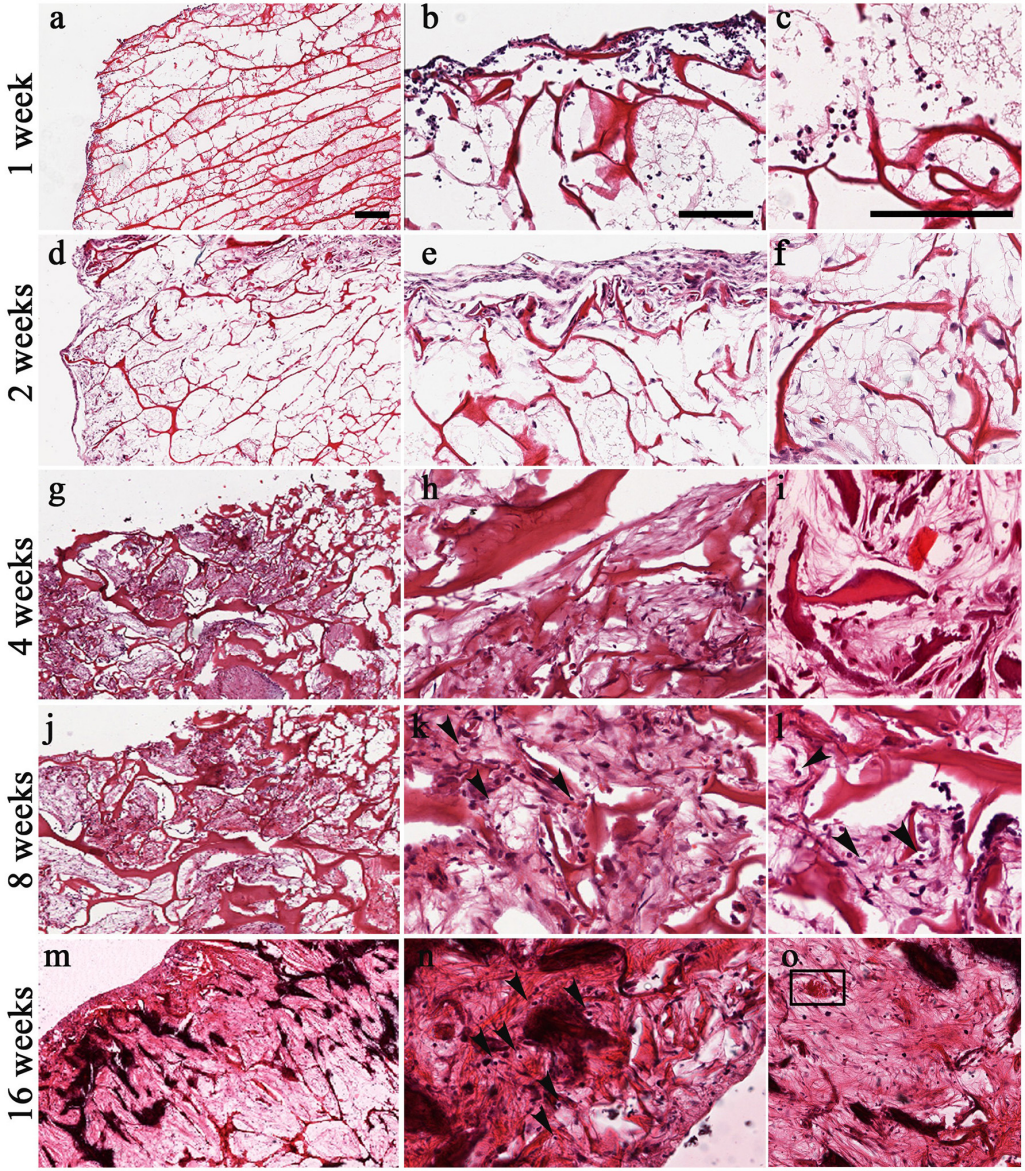
Time-course of chondrogenesis and angiogenesis within the implanted scaffolds as seen by H&E at 1 week **(a–c)**, 2 weeks **(d–f)**, 4 weeks **(g–i)**, 8 weeks **(j–l)**, and 16 weeks **(m–o)**. The formation of chondromyxoid-like tissue is evident at 2–16 weeks in the extracellular matrix **(d–o)**. Mesenchymal stem cells start to condensate and differentiate into chondrocytes. Arrows in **(k,l,n)** indicate the presence of pericellular lacunae resembling chondrocytes. Square in **(o)** indicate blood vessel. Scale bars: 200 μm for all images.

H&E staining confirmed the tolerability of the scaffold, already shown previously (Calabrese et al., 2016b), as revealed by the lack of necrotic areas and the slight and transitory inflammatory reaction seen sometimes in the explanted scaffolds (Figure 3). In fact, only at the first 2 weeks after implantation it was possible to see a small number of granulocytes, which are specific elements of inflammation (Figures 3a–f) that disappeared over time (Figures 3g–o).

H&E staining also showed the capacity of the scaffold to promote the formation of new chondral tissue, as demonstrated by the capacity of the biomaterial to recruit host cells that infiltrate, adhere and grow into the scaffold. Although host cells were confined in the surface during the first 2 weeks (Figures 3b,e), they started to infiltrate also the inner part of the scaffold at week 4 (Figures 3g–o). Furthermore, H&E staining showed a gradual increase of cartilage-like tissue within the pores of the scaffold starting at 4 weeks after implantation, as demonstrated by the presence of chondromyxoid-like matrix (Figures 3h,k,n). Interestingly, chondrocyte-like cells and pericellular lacunae, which are typical cartilage structures, were visible at 8 and 16 weeks after implantation (Figures 3k,l,n, arrows).

H&E staining also confirmed the FMT findings concerning the new vascularization of the scaffold by displaying the presence of structures resembling blood vessels (insert in Figure 3o).

**Figure 4:**
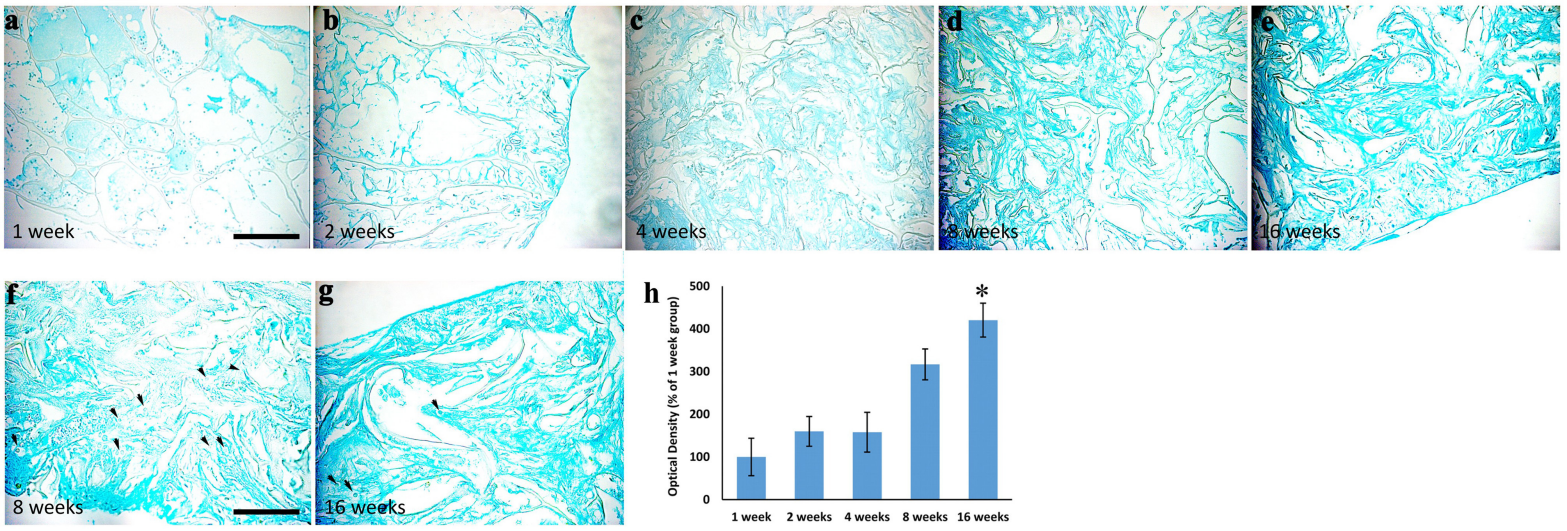
Alcian Blue staining showing the time-course of extracellular matrix deposition within the scaffolds implanted and explanted at 1 **(a)**, 2 **(b)**, 4 **(c)**, 8 **(d)**, and 16 **(e)** weeks after surgery, magnification 20x. It is evident that extracellular matrix deposition increased overtime. Arrows in **(f,g)** indicate the presence of pericellular lacunae, typical structures of cartilage, magnification 40x. The graph **(h)** shows the analysis of optical density relative to the Alcian Blue staining showed in **(a–e)**. A three- and four-fold increase of the staining density is evident at 8 and 16 weeks, respectively. Asterisk indicates significant difference from 1week group. Scale bars, in **a**, for **(a–e)**: 100 μm; in **f** for **(f–g)**: 50 μm.

**Figure 5:**
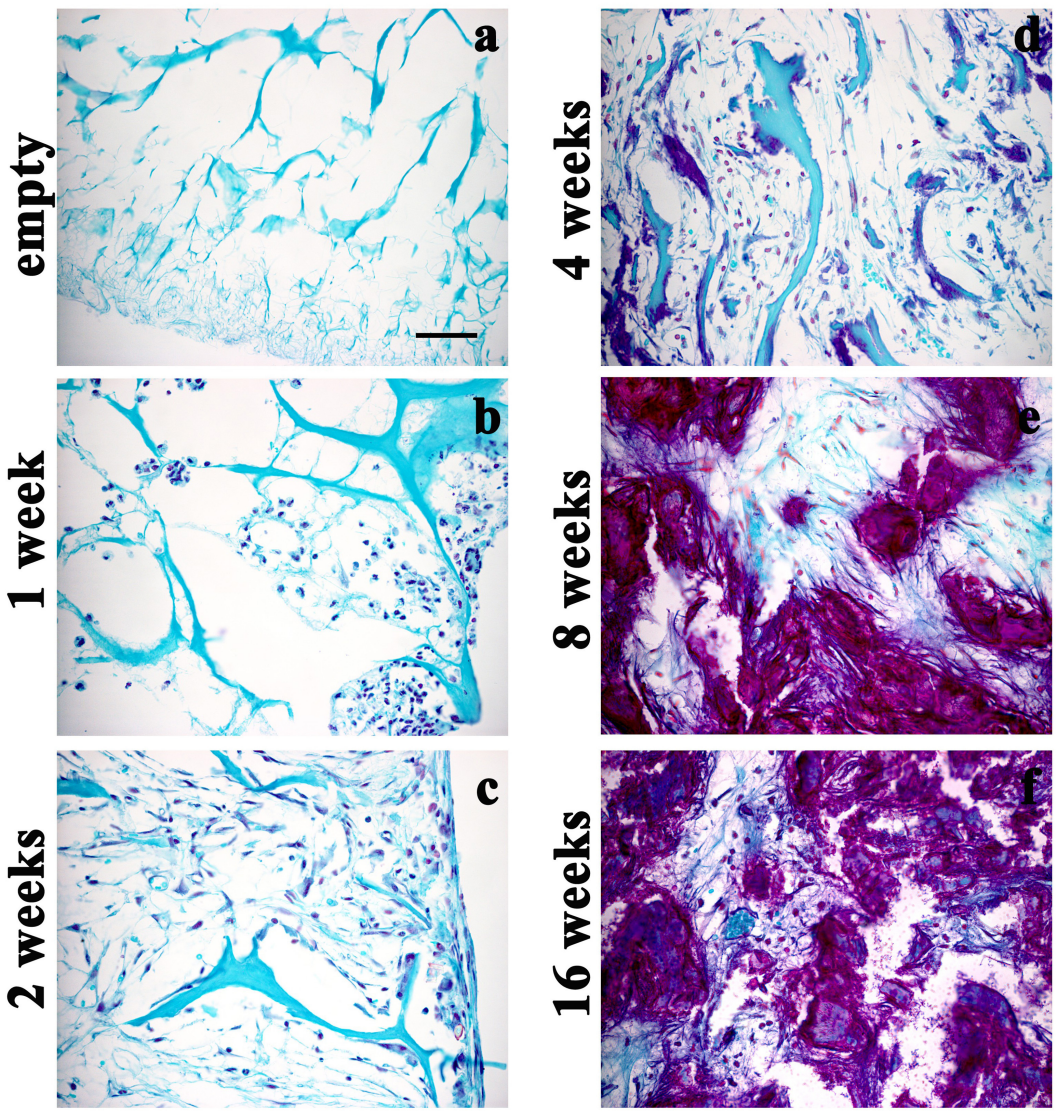
Safranin O/Fast green staining displaying the time-course of GAGs within the scaffolds either not implanted **(a)** or implanted and then explanted at 1 **(b)**, 2 **(c)**, 4 **(d)**, 8 **(e)**, and 16 **(f)** weeks after surgery, magnification 40x. The GAGs of newly formed cartilage are increased overtime. Scale bar in **a**: 50 μm.

These data were further sustained by the Alcian Blue staining (Figure 4), which revealed the presence of spindled and stellate mesenchymal cells as early as 2 weeks after implantation (Figure 4b). Then, the amount of extracellular matrix increases gradually (Figures 4c–d) and became strong at 16 weeks (Figure 4e), confirming the cartilage-like feature of the newly formed tissue. OD quantification confirms the gradual increase of cartilage-like matrix (ANOVA: *P* < 0.05; Figure 4a) that became significantly higher at 16 weeks, compared to average levels at 1 week (Tukey's *post hoc* test: *P* < 0.05, Figure 4a). To further reveal the cartilaginous nature of the freshly produced tissue within the scaffold, we assessed a Safranin O/ Fast green staining (Figures 5a–f). The red staining showed the GAGs of newly formed cartilage, while the green staining displayed collagens. In accordance with Alcian Blue, Safranin O/Fast green staining displayed a gradual increase of cartilage-like matrix overtime that became markedly more evident at 8 and 16 weeks (Figures 5e,f).

**Figure 6:**
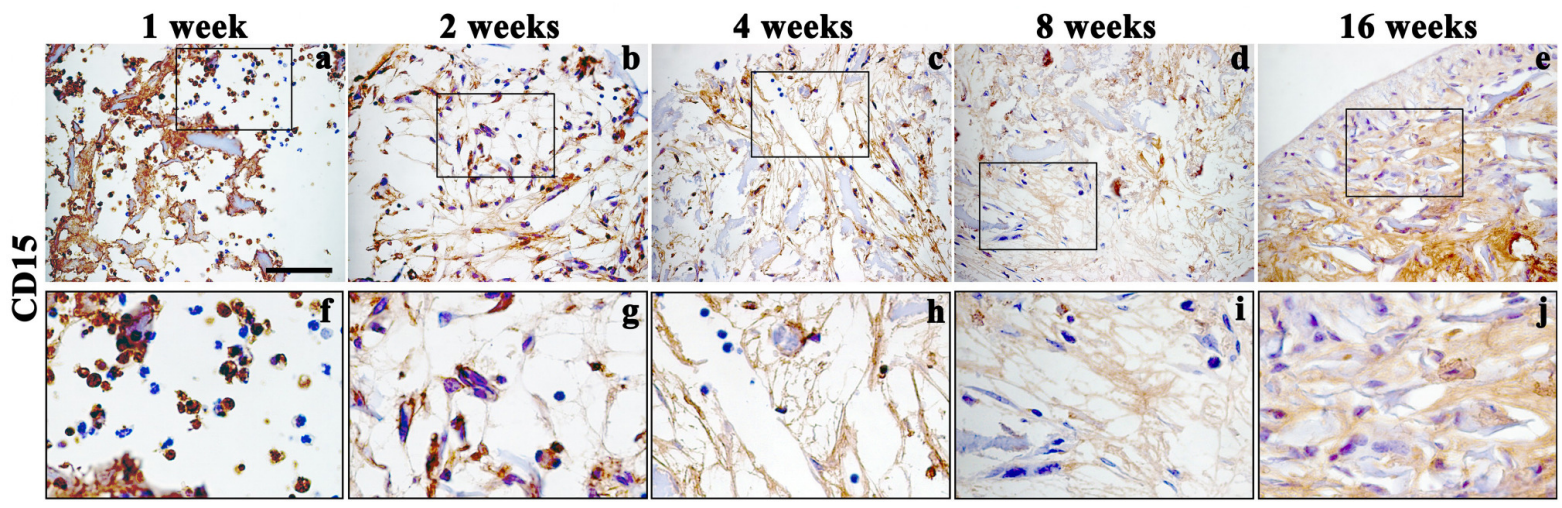
Immunohistochemistry analysis of inflammatory marker CD15 assessed on explanted scaffolds at 1 (**a**, enlarged in **f**), 2 (**b**, enlarged in **g**), 4 (**c**, enlarged in **h**), 8 (**d**, enlarged in **i**), and 16 (**e**, enlarged in **j**) weeks, magnification 40x. Scale bars: 50 μm.

**Figure 7:**
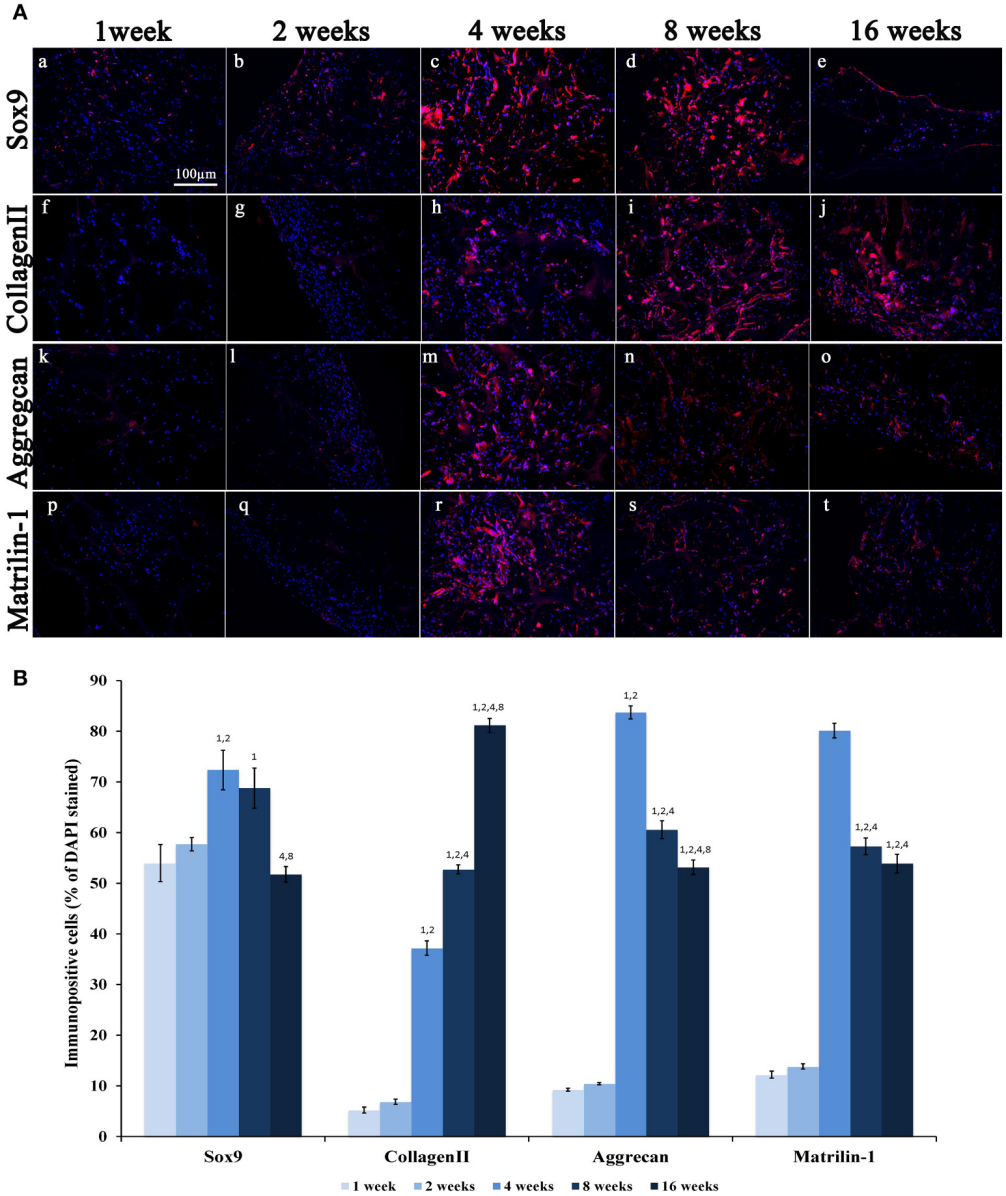
**(A)** Immunofluorescence analysis of representative chondrogenic markers:(a–e) Sox9, (f–j) CollagenII, (k–o) Aggrecan and (p–t) Matrilin-1, performed on collagen-based scaffolds after 1, 2, 4, 8, and 16 weeks post-implantation, magnification 20x. Scale bars: 100 μm for all fluorescence images. **(B)** Average cellular positivity for chondrogenic markers. Percentage of Sox9, CollagenII, Aggrecan, and Matrilin-1 are calculated on the total number of DAPI stained cells in the investigated fields.

Moreover, to further confirm both the biocompatibility and the capacity of biomimetic 3D scaffold to promote chondrogenesis, we performed immunohistochemical analysis using specific markers of inflammation and cartilage, such as CD15 and Sox9, type-II Collagen, Aggrecan and Matrilin-1 respectively, on histological sections obtained at 1, 2, 4, 8, and 16 weeks post-grafting (Figures 6, 7).

In accordance with H&E staining, immunohistochemical analysis of CD15, a granulocytes marker, showed a signal only during the first 2 weeks, (Figures 6a,b enlarged in Figures 6f,g) after implantation, confirming the presence of a temporary inflammatory process that missing over time (Figures 6 c–e enlarged in Figures 6h–j).

A weak expression of Sox9 was seen during the first 2 weeks (Figures 7Aa,b,B) followed by a significant increase at weeks 4 and 8 (Figures 7Ac,d,B; ANOVA: *P* < 0.05; Tukey's HSD *post hoc* test: *P* < 0.05) and an evident reduction at week 16 (Figures 7Ae,B; Tukey's HSD *post hoc* test: *P* < 0.05). Type-II Collagen displayed a very weak staining during the first 2 weeks (Figures 7Af,g,B), but a more robust signal appeared after 4 weeks (Figures 7Ah–j,B; ANOVA: *P* < 0.05; Tukey's HSD *post hoc* test: *P* < 0.05) and increased further at 8 and 16 weeks (Tukey's HSD *post hoc* test: *P* < 0.05). The expression of Aggrecan was very low during the first 2 weeks (Figures 7Ak,l,B), and then it reaches a peak at week 4 (Figure 7B; ANOVA: *P* < 0.05; Tukey's HSD *post hoc* test: *P* < 0.05) and then significantly and progressively decreased at weeks 8 and 16 (Figures 5b, 7Am–o; Tukey's HSD *post hoc* test: *P* < 0.05). As evident in Figures 7Ap–t,B, the Matrilin-1 expression pattern was similar to that of Aggrecan.

**Figure 8:**
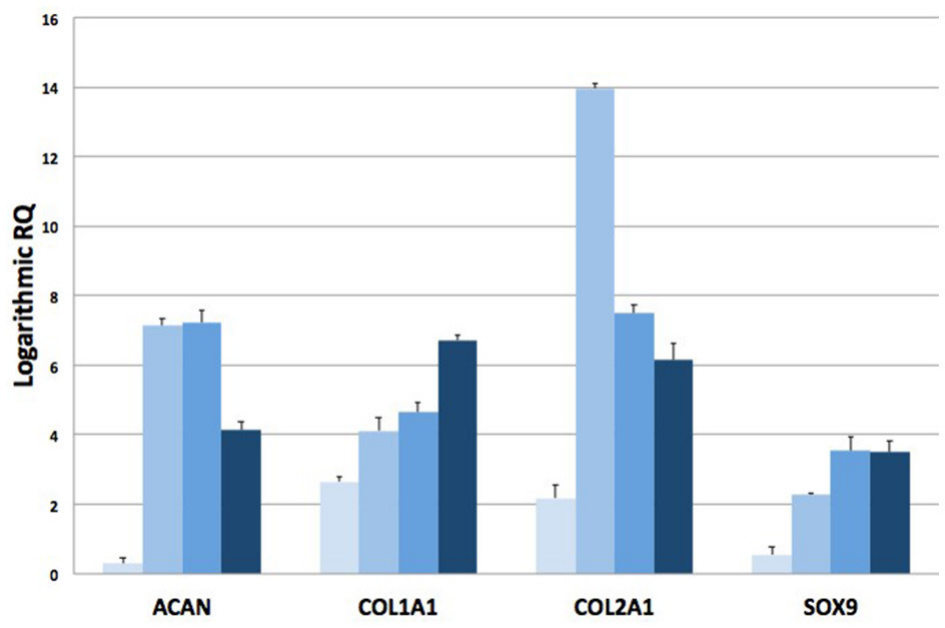
RT-qPCR analysis. Plots of logarithmic RQ values showing the time-course of the expression of chondrogenic genes at 1, 2, 4, 8, and 16 weeks after implantation.

RT-qPCR analysis has been also assessed on total RNA isolated from explanted scaffolds to quantify the expression of target genes related to chondral phenotype. The expression levels of chondrogenic genes, such as Type I Collagen (COL1A1), Type II Collagen (COL2A1), Aggrecan (ACAN) and Sox9, at 2, 4, 8, and 16 weeks have been compared to mRNA levels at week 1. Logarithmic RQ values are reported in Figure 8. COL1A1 shows an increased expression of almost three orders of magnitude during the second week that increased up to seven to 16 weeks. COL2A1 shows a distinctive peak of expression at week 4 (14 order of magnitude) with a subsequent decrease and reduced modulation (6 order of magnitude) until week 16. Sox9 mRNA concentration increases during the whole period with a massive regulation up to 3,5 order of positive modulation during the 16th week. ACAN shows a smallest expression during week 2 with peaks of positive regulation during the 4th and 8th week and a subsequent decrease modulation to week 16.

## Discussion

Several types of biomaterial, either of natural or synthetic origin, have been already used as tools for cartilage engineering (Vinatier et al., 2007, 2009; Zeugolis et al., 2008; Eglin et al., 2010; Solorio et al., 2013; Dahlin et al., 2014; Tsai et al., 2015; He et al., 2016; Hung et al., 2016; Raftery et al., 2016). Scaffolds are three-dimensional structure capable of supporting survival, migration, propagation and differentiation of cells as well as the formation of the extracellular matrix. Stimuli simulating the *in vivo* cartilage nature are required for chondrocyte differentiation. We have recently showed that a collagen-hydroxyapatite biomaterial is capable to promote human MSCs osteogenic differentiation both *in vitro* (Calabrese et al., 2016a) and *in vivo* (Calabrese et al., 2016b, 2017b). Moreover, the results achieved from previous *in vitro* studies have indicated that the biomaterial itself is able to stimulate chondrogenic differentiation of hADSCs seeded on it, but this process was slow and unable to be completed, thus causing cell death (Calabrese et al., 2017a). Moreover, the presence of chondrogenic bioactive factors in the culture medium strongly stimulates the differentiation process and the formation of an extracellular matrix having a chondromyxoid-like nature. Furthermore, cells embedded in the matrix were delimited by pericellular lacunae, typical structures of mature cartilage (Calabrese et al., 2017a). Given these *in vitro* results, whether this biomaterial could work *in vivo* without the need of growth factors and exogenous cells previously seeded in the scaffold was considered a fundamental prerequisite for clinical applications.

Here, we sought to verify if a cell-free, collagen-based 3D scaffold subcutaneously implanted in the mouse could be populated by resident cells and if the endogenous trophic support could be enough to commit these cells and induce the formation of cartilage-like matrix within the implanted biomaterial. The results have confirmed this hypothesis. Importantly, the biomaterial is well tolerated by immunocompetent animals as also shown in previous *in vivo* studies carried out with a collagen-hydroxyapatite biomaterial (Calabrese et al., 2016b, 2017b), since only a weak and transient granulocyte proliferation was seen during such a long *in vivo* observation. Moreover, the material has shown a high biocompatibility. In fact, the scaffolds have been wrapped by the host tissue and invaded by host cells. Angiogenic activity has been visualized both *in vivo* by FMT and after histology, which showed the formation of some blood vessels near the surface of the scaffolds. The vascularization of the implanted material, which is absent in the inner part of the scaffold, as also occurred in the natural hyaline cartilage, represents a sign of a successful integration of the graft into the host environment, together with the absence of significant inflammatory reactions. The presence of peripheral vascularization could in fact be beneficial for the trophic support of the cell population invading the scaffold. However, it is known that a successful chondrogenic process could be hampered by the vascularization of the matrix (Choi et al., 2010; Centola et al., 2013). An initial host reaction including inflammation and angiogenesis could be expected, especially given the ectopic implantation of the scaffolds in our experiments, but the reduction of angiogenic processes at the later time-points seen by FMT, as well as the absence of blood vessels in the inner part of the scaffold is promising and probably represents one of the factor promoting the observed chondrogenic maturation. H&E and Alcian Blue staining of the explanted biomaterial have shown that a relevant augmentation of chondral tissue occurred within the scaffold during a period of 16 weeks after subcutaneous implantation in the dorsum of mice. Histological analysis revealed that the material has been populated by host cells able to penetrate, adhere and grow into the scaffold. Although these cells were confined near the surface of the scaffold during the first 2 weeks after implantation, they were then able to migrate toward the inner part at the longer time-points, and give rise to the formation of chondromyxoid-like matrix including chondrocyte-like cells and pericellular lacunae, which are typical cartilage structures. Although several studies have tested the properties of cellularized scaffolds with concomitant use of trophic factors (Crawford et al., 2009; Zhang et al., 2013; Huang et al., 2016), fewer but promising studies including this one, have attempted to achieve cartilage formation within different biomaterials, without the use of exogenous cells or growth factors (Siclari et al., 2012; Lebourg et al., 2014). The chondrogenic differentiation has been confirmed by the expression of specific markers such as Sox9, type II Collagen, Aggrecan and Matrilin-1. Sox9 is a transcription factor that plays a key role in chondrogenesis, by controlling the expression of type II Collagen and Aggrecan as well as by supporting chondrocyte survival and hypertrophy (Ikegami et al., 2011; Lefebvre and Dvir-Ginzberg, 2017). Type-II Collagen is the unique type of collagen in hyaline cartilage and represents more than 50% of all protein. Aggrecan is the major proteoglycan in the articular cartilage and, together with type-II Collagen, represents the principal structural element of cartilage. Therefore, the observed increase of both Collagen II and Aggrecan at the longest time points is likely a cellular evidence of cartilage maturation in our mouse model. Matrilin-1 is a protein involved in the formation of filamentous networks in the cartilage extracellular matrix, by interacting with both type-II Collagen and Aggrecan (Muir, 1995; Lefebvre and Dvir-Ginzberg, 2017). Interestingly, Matrilin-1 could also act as an inhibitor of neovascularization (Foradori et al., 2014) so, its role could be relevant in our model, since its expression increased at 4 weeks after scaffold implantation and remained significantly high until 4 months, being also inversely correlated with the amount of AngioSense probe revealed *in vivo* by FMT. It seems therefore likely that the expression of Matrilin-1 could represent a spontaneous trigger for cartilage augmentation within the scaffold (Foradori et al., 2014).

In conclusion, our data suggest that this novel biomimetic material (i) is well tolerated *in vivo*, (ii) that it could successfully integrate to the host tissue and (iii) promote the formation of new cartilage-like tissue by recruiting the host cells and commit them to chondrogenic differentiation. The suitability of this material for supporting cartilage repair without using exogenous cells or growth factor is promising and represents a possible tool for human application of cartilage tissue engineering and repairing approaches.

## Author contributions

All authors had substantial contribution to the present work. Specifically, GC conceived the project, designed and performed the experiments and wrote the paper; RoG participated to conceive the project and perform the experiment and wrote the paper; RaG performed the immunohistochemistry experiments; SF executed the statistical analysis; EF and CF supplied the scaffolds and contributed to their construction and characterization; LS have done histology, microscopy and have participate to data analysis; LM supplied the surgical sample for immunohistochemistry experiments; MG participated to project preparation, data interpretation and manuscript writing; RP conceived the project, designed and performed the experiments, wrote the paper and she is also the corresponding author. All the authors gave the final approval of the version to be published.

### Conflict of interest statement

The authors declare that the research was conducted in the absence of any commercial or financial relationships that could be construed as a potential conflict of interest.
